# Immune Cell-Associated Protein Expression Helps to Predict Survival in Muscle-Invasive Urothelial Bladder Cancer Patients after Radical Cystectomy and Optional Adjuvant Chemotherapy

**DOI:** 10.3390/cells10010159

**Published:** 2021-01-15

**Authors:** Helge Taubert, Markus Eckstein, Elena Epple, Rudolf Jung, Katrin Weigelt, Verena Lieb, Danijel Sikic, Robert Stöhr, Carol Geppert, Veronika Weyerer, Simone Bertz, Astrid Kehlen, Arndt Hartmann, Bernd Wullich, Sven Wach

**Affiliations:** 1 Department of Urology and Pediatric Urology, University Hospital Erlangen, FAU Erlangen-Nürnberg, 91054 Erlangen, Germany; elena.epple@fau.de (E.E.); Katrin.Weigelt@uk-erlangen.de (K.W.); Verena.Lieb@uk-erlangen.de (V.L.); danijel.sikic@uk-erlangen.de (D.S.); Bernd.Wullich@uk-erlangen.de (B.W.); sven.wach@uk-erlangen.de (S.W.); 2 Institute of Pathology, University Hospital Erlangen, FAU Erlangen-Nürnberg, 91054 Erlangen, Germany; markus.eckstein@uk-erlangen.de (M.E.); Rudolf.Jung@uk-erlangen.de (R.J.); robert.stoehr@uk-erlangen.de (R.S.); carol.geppert@uk-erlangen.de (C.G.); veronika.weyerer@uk-erlangen.de (V.W.); simone.bertz@uk-erlangen.de (S.B.); Arndt.Hartmann@uk-erlangen.de (A.H.); 3 Institute of Medical Microbiology, Medical School, Martin-Luther-University Halle-Wittenberg, 06112 Halle (Saale), Germany; astrid.kehlen@uk-halle.de

**Keywords:** CCL2, CD68, CD163, bladder cancer, immune cells, chemotherapy, tumor stage, nodal stage

## Abstract

**Simple Summary:**

Adjuvant chemotherapy following radical cystectomy is a common therapy for muscle invasive bladder cancer (MIBC) patients. No applicable biomarkers exist to predict which patients will benefit from chemotherapy. Three immune cell markers, the chemokine CC motif ligand 2 (CCL2), the pan macrophage marker CD68, and the M2 macrophage marker CD163, were examined using immunohistochemistry to determine their predictive value for chemotherapy responses in different nodal stage and tumor stage subgroups. The presence of tumor-infiltrating immune cells, characterized by the markers CD68, CD163, and CCL2, was associated with a superior prognosis, and chemotherapy may not add an advantage for prognosis. However, a depleted immune microenvironment, here represented as a reduction or loss of macrophages, helped to predict the benefit of chemotherapy in N1 + 2 stage patients. Altogether, it is meaningful to consider the abundance of immune cells, such as macrophages, to better predict the response to chemotherapy for bladder cancer (BCa) patients after radical treatment.

**Abstract:**

Bladder cancer (BCa) is the tenth most commonly diagnosed malignant cancer worldwide. Although adjuvant chemotherapy following radical cystectomy is a common therapy for muscle invasive bladder cancer patients, no applicable biomarkers exist to predict which patients will benefit from chemotherapy. In this study, we examined three immune cell markers, the chemokine CC motif ligand 2 (CCL2), the pan macrophage marker cluster of differentiation 68 (CD68) and the M2 macrophage marker cluster of differentiation 163 (CD163), using immunohistochemistry to determine their predictive value for the chemotherapy response in different nodal stage (pN0 vs. pN1 + 2) and tumor stage subgroups (pT2 vs. pT3 + 4). The prognosis was studied in terms of the overall survival (OS), disease-specific survival (DSS), and recurrence-free-survival (RFS) in 168 muscle invasive BCa patients. Chemotherapy was associated with a poorer prognosis in patients with a higher expression of the immune markers CCL2 (RFS), CD68 (DSS and RFS), and CD163 (DSS and RFS) in the N0 group and with poorer survival in patients with a higher expression of the immune markers CCL2 (OS, DSS, and RFS), CD68 (OS, DSS, and RFS), and CD163 (OS, DSS, and RFS) in the pT2 group when compared with treatments without chemotherapy. In contrast, chemotherapy was associated with a better prognosis in patients with a low expression of the immune markers CCL2 (DSS and RFS), CD68 (OS, DSS, and RFS), and CD163 (OS) in the N1 + 2 group. In addition, chemotherapy was associated with improved survival in patients with a low expression of the immune marker CD68 (OS and DSS) and there was a trend for a better prognosis in patients with a low expression of CD163 (OS) in the pT3 + 4 group compared to patients not treated with chemotherapy. Interestingly, CD68 appeared to be the most applicable immune marker to stratify patients by the outcome of chemotherapy in the nodal stage and tumor stage groups. Overall, we suggest that, in addition to the clinical factors of tumor stage and nodal stage, it is also meaningful to consider the abundance of immune cells, such as macrophages, to better predict the response to chemotherapy for BCa patients after radical treatment.

## 1. Introduction

Urothelial bladder cancer (BCa) accounts for approximately 3% of global cancer diagnoses. It was recently the 10th most commonly diagnosed cancer and the 13th leading cause of cancer-related death worldwide [1]. BCa originates from the urothelium of the ureter (upper urinary tract), urethra, and bladder [1,2]. Approximately 25% of BCa cases are muscle-invasive BCa (MIBC) cases [3]. The current therapy for MIBC consists of radical cystectomy, with bilateral lymphadenectomy in combination with platinum-based perioperative chemotherapy in patients with extravesical tumors and/or lymph node metastasis. It is well accepted that the degree of lymph node involvement and tumor stage are prognostic factors for MIBC [4,5]. Adjuvant chemotherapy appeared to be effective in lymph node positive BCa patients regardless of their p53 status [6].

Others and we have shown that the tumor immune microenvironment is also associated with survival [7,8,9,10,11,12]. In particular, the presence of tumor infiltrating immune cells, i.e., leukocytes, including lymphocytes identified by their protein or gene expression profile, is associated with superior 5-year overall survival (OS) or disease-specific survival (DSS) [7,9,12]. Recently, we found that the expression of the immune cell marker chemokine CC motif ligand 2 (CCL2) was differentially associated with prognosis depending on the lymph node stage. In the N0 group, CCL2 positivity in immune cells (ICs) was a positive independent prognostic factor for OS, DSS, and recurrence-free survival (RFS), whereas, in the N1 + 2 group, CCL2 positivity was a negative independent factor for OS and RFS [13]. Based on this finding, we were interested in whether the expression of immune cell markers could be helpful in the prediction of the chemotherapy treatment response in MIBC patients stratified by their nodal stage or tumor stage.

In this study, we examined three immune cell markers: CCL2, the pan (M1 + M2) macrophage marker CD68, and the M2 macrophage marker CD163 for their value for predicting the response to adjuvant chemotherapy. While M1 macrophages are considered tumor-inhibiting, M2 macrophages instead appear as tumor-promoting [14]. CCL2 has been shown to be expressed in tumor-associated macrophages [15,16,17]. In addition, we showed a strong correlation of CCL2 with the expression of programmed cell death 1 ligand 1 (PD-L1), which is expressed by several immune cells, such as T-cells, natural killer cells, macrophages, myeloid dendritic cells, and B-cells [13]. This finding suggests that CCL2 expression is not restricted to only macrophages but is also part of the concerted immune cell response.

CD68 (macrosialin) is a glycosylated type I transmembrane protein and a monocyte/pan macrophage marker [18,19]. CD163 is a scavenger receptor cysteine rich family type B member (hemoglobin scavenger receptor) and a monocyte/M2 macrophage marker [20,21]. Both CD68 and CD163 were examined for their association with DSS in MIBC [11]. However, both showed no association with DSS, but CD68 expression was able to modulate the positive association between CD3 expression and DSS. The authors found that the group with a CD3 high/CD68 low phenotype had the best prognosis and the group with a CD3 low/CD68 high phenotype had the poorest prognosis in MIBC patients [11].

Recently, we showed that higher CD68 and CCL2 staining in immune cells was associated with better DSS and RFS in MIBC patients, whereas CD163 expression was not associated with prognosis (OS, DSS, or RFS) [13]. Our working hypothesis is that the presence of tumor-infiltrating immune cells, here represented as macrophages characterized by the markers CD68, CD163, and CCL2, could be associated with a better prognosis, but that chemotherapy may not add an advantage for prognosis. However, a depleted immune microenvironment, here represented as a reduction or loss of macrophages, may help to predict a benefit of chemotherapy in patients with a general worse prognosis (N1 + 2 stage or T3/4 stage patients). However, no study has yet examined the value of CCL2, CD68, or CD163 expression for predicting the response to adjuvant chemotherapy in MIBC.

## 2. Results

### 2.1. Association of Chemotherapy Treatment with Prognosis in Different Nodal Stage and Tumor Stage Groups of Patients with Muscle-Invasive Bladder Cancer

**Figure 1:**
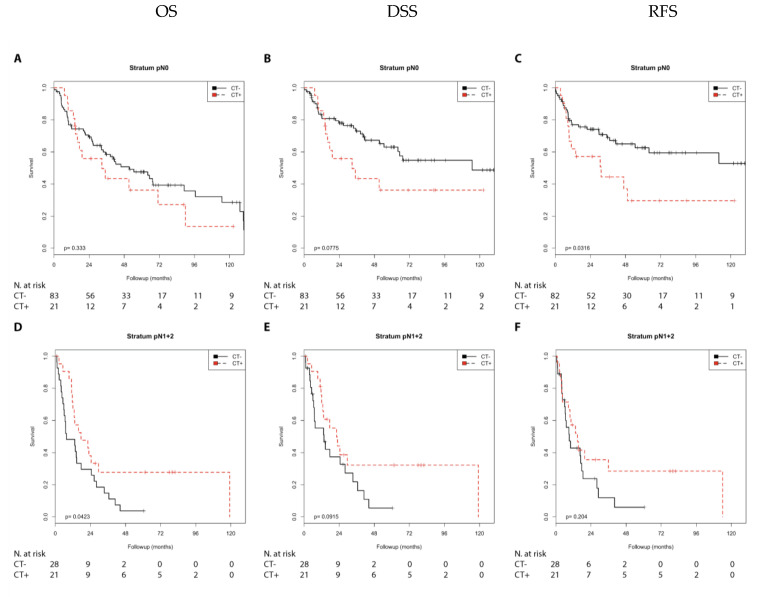
Kaplan–Meier analysis: Association of chemotherapy with prognosis (overall survival (OS), disease-specific survival (DSS), and recurrence-free-survival (RFS)) stratified by lymph node stage (N0 or N1 + 2). In the N0 group (**A**–**C**), chemotherapy was not associated with OS (**A**) but showed a trend of association with DSS (**B**; *p* = 0.078) and was associated with RFS (**C**; *p* = 0.032). In the N1 + 2 group (**D**–**F**), chemotherapy was associated with OS (**D**; *p* = 0.042), showed a trend of association with DSS (**E**; *p* = 0.091) and was not associated with RFS (**F**).

We studied a cohort of 168 MIBC patients (Supplementary Table S1 and Table S2A). Chemotherapy was associated with the tumor stage (r_s_ = 2.28; *p* = 0.003) and as a trend with the nodal stage (r_s_ = 0.150; *p* = 0.052; Supplementary Table S2B). The immune markers (CCL2, CD68, and CD163) were not associated with the tumor stage or nodal stage (Supplementary Table S2C). First, we investigated whether chemotherapy was associated with prognosis (OS, DSS, and RFS) after stratifying bladder cancer patients by lymph node stage (N0 vs. N1 + 2 vs. NX) and tumor stage (pT2 vs. pT3 + 4) in a Kaplan–Meier analysis (Supplementary Table S3). In the N0 group, chemotherapy was significantly associated with a shorter RFS (*p* = 0.032) and as trend with a shorter DSS (*p* = 0.078) when compared with no chemotherapy (Supplementary Table S3; Figure 1).

**Figure 2:**
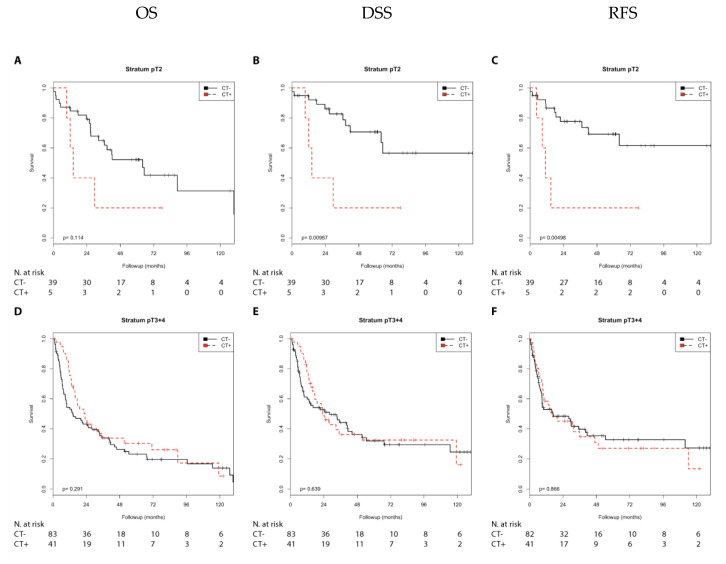
Kaplan–Meier analysis: Association of chemotherapy with prognosis (OS, DSS, and RFS) stratified by tumor stage (pT2 or pT3 + 4). In the pT2 group (**A**–**C**), chemotherapy was not associated with OS (**A**) but was associated with DSS (**B**; *p* = 0.010) and RFS (**C**; *p* = 0.005). In the pT3 + 4 group (**D**–**F**), chemotherapy was not associated with OS (**D**), DSS (**E**), or RFS (**F**).

In contrast, in the N1 + 2 group, chemotherapy was significantly associated with a better OS (*p* = 0.042) and showed a trend toward a better DSS (*p* = 0.091) compared with no chemotherapy (Figure 1). In the pT2 group, considering the low number of patients, chemotherapy was significantly associated with a poorer DSS (*p* = 0.010) and poorer RFS (*p* = 0.005) compared with no chemotherapy. However, in the pT3 + 4 group, there was no difference in prognosis (OS, DSS, or RFS) between patients treated with or without chemotherapy (Supplementary Table S3; Figure 2).

Next, we performed a univariate Cox’s regression analysis to characterize the relative risks associated with chemotherapy (Table 1). Comparable to the Kaplan–Meier analysis, in the N0 group, chemotherapy was significantly associated with a shorter RFS (relative risk (*RR*) = 2.03; *p* = 0.035) and as a trend with a shorter DSS (RR = 1.82; *p* = 0.082) (Table 1). In contrast, in the N1 + 2 group, chemotherapy was significantly associated with a better OS (RR = 0.52; *p* = 0.046) and showed a trend toward a better DSS (RR = 0.55; *p* = 0.097). In the pT2 group, considering the low number of patients, chemotherapy was significantly associated with a poorer DSS (RR = 4.10; *p* = 0.016) and a poorer RFS (RR = 4.61; *p* = 0.010) when compared with no chemotherapy. Again, in the pT3 + 4 group, there was no difference in prognosis (OS, DSS, or RFS) between patients treated with or without chemotherapy (Table 1).

In summary, an unfavorable prognosis was detected in the N0 (RFS) and pT2 (DSS and RFS) groups treated with chemotherapy, whereas a favorable effect on prognosis was observed in the N1 + 2 (OS) group treated with chemotherapy.

As both the tumor stage and nodal stage are associated with chemotherapy, we subdivided the patients into four groups, N0/T2, N0/T3 + 4, N1 + 2/T2, and N1 + 2/T3 + 4. In the Kaplan–Meier analysis in the N0/T2 group, chemotherapy was significantly associated with a shorter DSS (*p* = 0.012), a shorter RFS (*p* = 0.011), and as a trend with a shorter OS (*p* = 0.056; Supplementary Table S4). In the N1 + 2/T3 + 4 group, chemotherapy was significantly associated with a longer OS (*p* = 0.036), and as a trend with a longer DSS (*p* = 0.059; Supplementary Table S4).

In the univariate Cox’s regression analysis in the N0/T2 group, chemotherapy was significantly associated with a shorter DSS (RR = 10.28; *p* = 0.044), a shorter RFS (RR = 10.47; *p* = 0.042), and as a trend with a shorter OS (RR = 6.21; *p* = 0.096; Table 1). In the N1 + 2/T3 + 4 group, chemotherapy was significantly associated with a longer OS (RR = 0.48; *p* = 0.042), and as a trend with a longer DSS (RR = 0.48; *p* = 0.066).

In summary of the above mentioned results, an unfavorable effect of chemotherapy on prognosis was detected in the N0/T2 (DSS and RFS) group, whereas chemotherapy had a favorable effect on prognosis in the N1 + 2/T3 + 4 (OS) group.

Based on our previous results [9,13], we hypothesized that it would be possible to further substratify patients into groups using immune markers. We sought to identify patients with an N0 status and/or pT2 tumors that might benefit from not receiving chemotherapy. We also tried to identify patients with an N+ status and/or progressed pT3 + 4 tumors that would have a clinical benefit from chemotherapy.

### 2.2. Association of Chemotherapy Treatment with Prognosis in Different Nodal Stage and Tumor Stage Groups Stratified by CCL2, CD68, and CD163 Expression in Muscle Invasive Bladder Cancer Patients

A cohort of 168 muscle invasive BCa patients was studied for CCL2, CD68, and CD163 protein expression in immune cells using immunohistochemistry (IHC) as previously described ([13] and Supplementary Figure S1). The CCL2 expression was separated into negative (≤6% of the immune cells; *N* = 98) and positive (>6% of the immune cells; *N* = 70; Supplementary Table S1) groups, the CD68 expression was separated into low (≤10.4; *N* = 82) and high (>10.4; *N* = 86) groups, and the CD163 expression was separated into low (≤10.34; *N* = 84) and high (>10.34; *N* = 84) groups. The clinicopathological data of the muscle invasive BCa patients were previously reported [13] and are presented in Supplementary Table S1.

We examined the survival characteristics of patients receiving or not receiving chemotherapy in the two nodal stage groups (N0/N1 + 2) and in the two tumor stage groups (T2/T3 + 4) stratified by their CCL2, CD68, or CD163 expression in a Kaplan–Meier analysis and univariate Cox’s regression analysis (Supplementary Table S5, Table 2 and Table 3). For a better overview, we considered each immune marker (CCL2, CD68, or CD163) separately in the analyses.

#### 2.2.1. Immune Marker CCL2

CCL2 can be expressed in different immune cells. The association of its positive expression (>6%) with prognosis is dependent on the nodal stage. As previously reported, CCL2 expression can be associated with a superior prognosis (N0 stage) or with a poor prognosis (N1 + 2 stage) [13].

**Figure 3:**
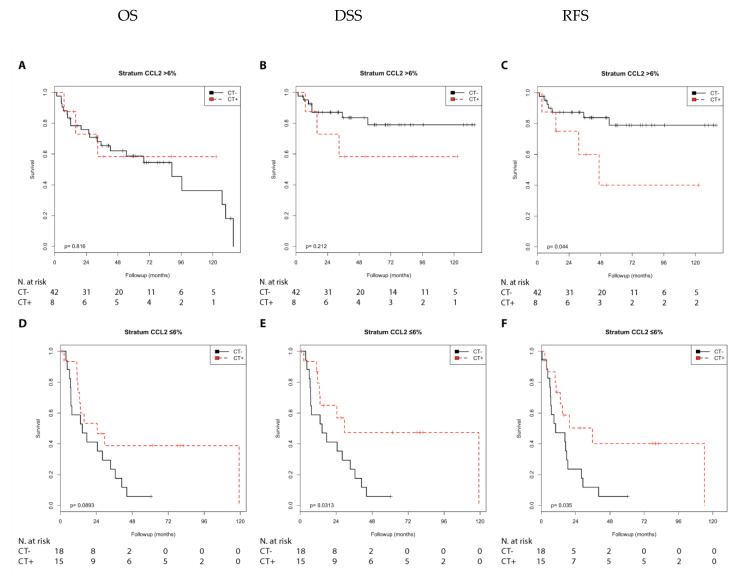
Kaplan–Meier analysis: Association of chemotherapy with prognosis (OS, DSS, and RFS) in the N0/N1 + 2 groups stratified by CCL2 expression (>6% or ≤6%). In the N0 group (**A**–**C**) with CCL2 (>6%), chemotherapy treatment was not associated with OS (**A**) or DSS (**B**) but was associated with shorter RFS (**C**; *p* = 0.044). In the N1 + 2 group (**D**–**F**) with CCL2 (≤6%), chemotherapy was not associated with OS (**D**) but was associated with longer DSS (**E**; *p* = 0.031) and longer RFS (F; *p* = 0.035).

**Figure 4:**
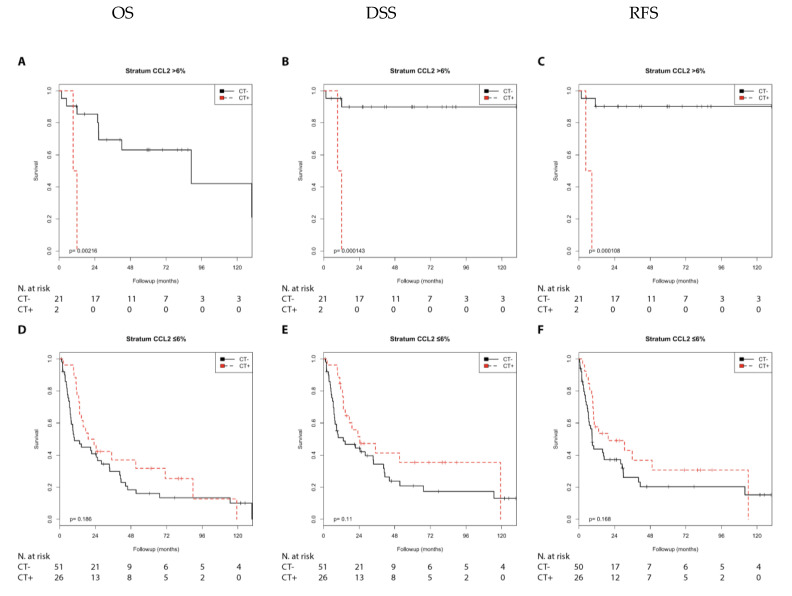
Kaplan–Meier analysis: Association of chemotherapy with prognosis (OS, DSS, and RFS) in the pT2/pT3 + 4 groups stratified by CCL2 expression (>6% or ≤6%). In the pT2 group (**A**–**C**) with CCL2 (>6%), chemotherapy was associated with shorter OS (**A**; *p* = 0.002), shorter DSS (**B**; *p* < 0.001), and shorter RFS (**C**; *p* < 0.001). In the pT3 + 4 group (**D**–**F**) with CCL2 (≤6%), chemotherapy was not associated with OS (**D**), DSS (**E**), or RFS (**F**).

We first performed a Kaplan–Meier analysis. Chemotherapy was associated with a shorter RFS (*p* = 0.044) than no chemotherapy in the N0 group with CCL2 positivity. In contrast, chemotherapy treatment was associated with a longer DSS (*p* = 0.031), a longer RFS (*p* = 0.035), and with a trend toward a longer OS (*p* = 0.089) compared with no chemotherapy in the N1 + 2 group with negative CCL2 expression (Supplementary Table S5; Figure 3). Furthermore, chemotherapy was associated with a shorter OS (*p* = 0.002), DSS, and RFS (both *p* < 0.001) compared with no chemotherapy in the pT2 group with positive CCL2 expression (Figure 4). In addition, chemotherapy was associated with a shorter RFS (*p* = 0.026) in the pT3 + 4 group with positive CCL2 expression (Supplementary Table S5).

Next, we performed univariate Cox’s regression analysis to characterize the relative risks associated with chemotherapy (Table 2 and Table 3). Chemotherapy was only associated as trend with a shorter RFS (RR = 3.33; *p* = 0.057) when compared with no chemotherapy in the N0 group with CCL2 positivity. In contrast, chemotherapy was associated with a longer DSS (RR = 0.39; *p* = 0.038), a longer RFS (RR = 0.41; *p* = 0.041), and as trend toward a longer OS (RR = 0.50; *p* = 0.096) compared with no chemotherapy in the N1 + 2 group with negative CCL2 expression (Table 2).

In addition, chemotherapy was associated with a shorter OS (RR = 11.56; *p* = 0.015), DSS (RR = 24.61; *p* = 0.009), and RFS (RR = 25.51; *p* = 0.009) compared with no chemotherapy in the pT2 group with positive CCL2 expression. Furthermore, chemotherapy was associated with a shorter RFS (RR = 2.52; *p* = 0.032) in the pT3 + 4 group with positive CCL2 expression (Table 3).

#### 2.2.2. Immune Marker CD68

CD68 is a pan macrophage marker. The association of its expression with prognosis was also dependent on the nodal stage. In the N0 patient group, high CD68 staining was significantly associated with a longer DSS and RFS compared with low CD68 staining (*p* = 0.023 and *p* = 0.016, respectively), but no association was seen in the N1 + 2 group [13].

**Figure 5:**
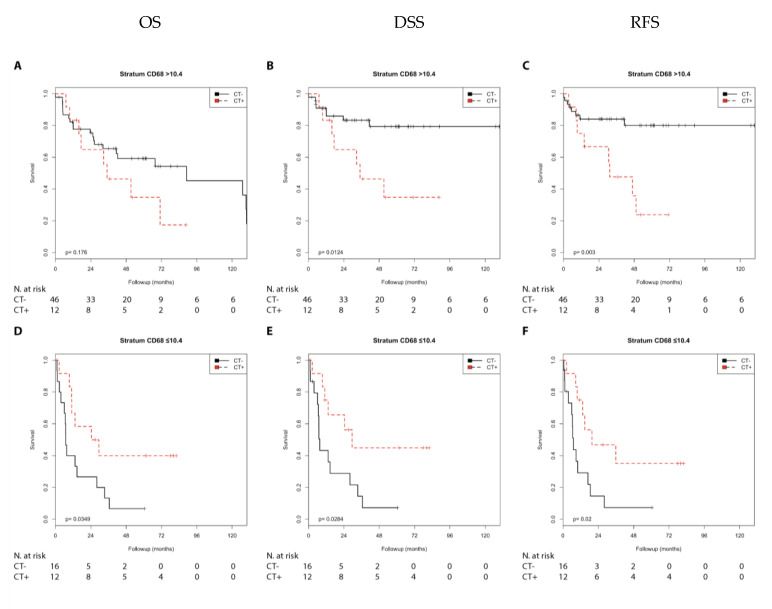
Kaplan–Meier analysis: Association of chemotherapy with prognosis (OS, DSS, and RFS) in the N0/N1 + 2 groups stratified by CD68 expression (>10.4 or ≤10.4). In the N0 group (**A**–**C**) with CD68 (>10.4), chemotherapy was not associated with OS (**A**) but with shorter DSS (**B**; *p* = 0.012) and shorter RFS (**C**; *p* = 0.003). In the N1 + 2 group (**D**–**F**) with CD68 (≤10.4), chemotherapy was associated with longer OS (**D**; *p* = 0.035), longer DSS (**E**; *p* = 0.028), and longer RFS (**F**; *p* = 0.020).

In the Kaplan–Meier analysis, chemotherapy was associated with a shorter DSS (*p* = 0.012) and RFS (*p* = 0.003) compared with no chemotherapy in the N0 group with high CD68 expression. In contrast, chemotherapy was associated with a longer OS (*p* = 0.035), DSS (*p* = 0.028), and RFS (*p* = 0.020) in the N1 + 2 group with low CD68 expression (Supplementary Table S2; Figure 5).

**Figure 6:**
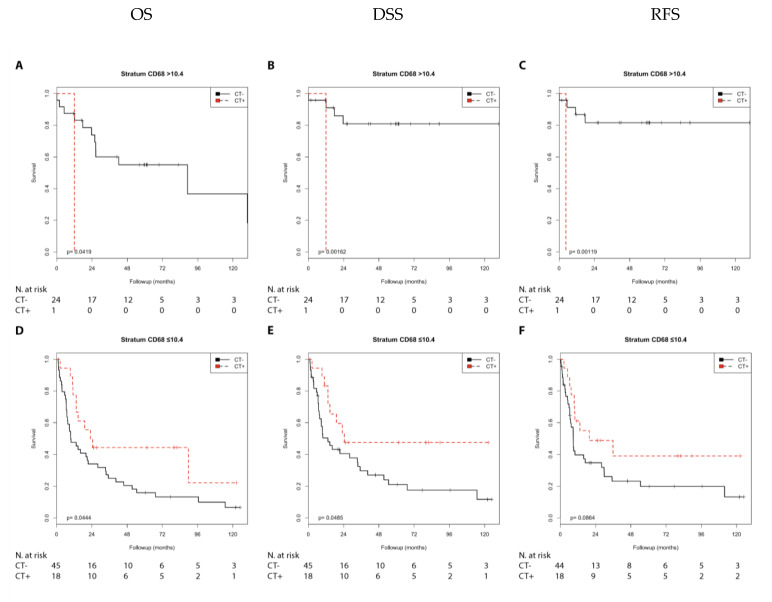
Kaplan–Meier analysis: Association of chemotherapy with prognosis (OS, DSS, and RFS) in the pT2/pT3 + 4 groups stratified by CD68 expression (>10.4 or ≤10.4). In the pT2 group (**A**–**C**) with CD68 (>10.4), chemotherapy was associated with shorter OS (**A**; *p* = 0.042), shorter DSS (**B**; *p* = 0.002), and shorter RFS (**C**; *p* = 0.001). In the pT3 + 4 group (**D**–**F**) at CD68 (≤10.4), chemotherapy was associated with longer OS (**D**; *p* = 0.044), longer DSS (**E**; *p* = 0.048), and as trend with longer RFS (**F**; *p* = 0.086).

Chemotherapy was associated with a shorter OS (*p* = 0.042), DSS (*p* = 0.002), and RFS (*p* = 0.001) compared with no chemotherapy in the pT2 group with high CD68 expression. In addition, chemotherapy was associated with a shorter RFS (*p* = 0.027) in the pT3 + 4 group with high CD68 expression. In contrast, chemotherapy was associated with a longer OS (*p* = 0.044) and DSS (*p* = 0.048) and showed a trend with a longer RFS (*p* = 0.086) in the pT3 + 4 group with low CD68 expression (Supplementary Table S5; Figure 6)

Next, we performed a univariate Cox’s regression analysis. Chemotherapy was associated with a shorter DSS (RR = 3.38; *p* = 0.019) and RFS (RR = 3.97; *p* = 0.006) compared with no chemotherapy in the N0 group with high CD68 expression. In contrast, chemotherapy was associated with a longer OS (RR = 0.39; *p* = 0.042), DSS (RR = 0.35; *p* = 0.036), and RFS (RR = 0.34; *p* = 0.026) in the N1 + 2 group with low CD68 expression (Table 2).

Chemotherapy was associated with a shorter DSS (RR = 21.91; *p* = 0.029), RFS (RR = 22.98; *p* = 0.027), and as a trend with OS (RR = 7.39; *p* = 0.083) compared with no chemotherapy in the pT2 group with high CD68 expression. In addition, chemotherapy was associated with a shorter RFS (RR = 2.14; *p* = 0.031) in the pT3 + 4 group with high CD68 expression. In contrast, chemotherapy was associated with a longer OS (RR = 0.51; *p* = 0.049), and a trend with a longer DSS (RR = 0.48; *p* = 0.053) and RFS (RR = 0.54; *p* = 0.091) in the pT3 + 4 group with low CD68 expression (Table 3).

#### 2.2.3. Immune Marker CD163

CD163 is an M2 macrophage marker, and M2 macrophages are considered to have antiapoptotic effects [20,22]. In the N1 + 2 group, high CD163 expression was significantly associated with a shorter OS (*p* = 0.016) compared with low CD163 expression and showed a trend with DSS (*p* = 0.086); however, no association was found in the N0 group [13].

**Figure 7:**
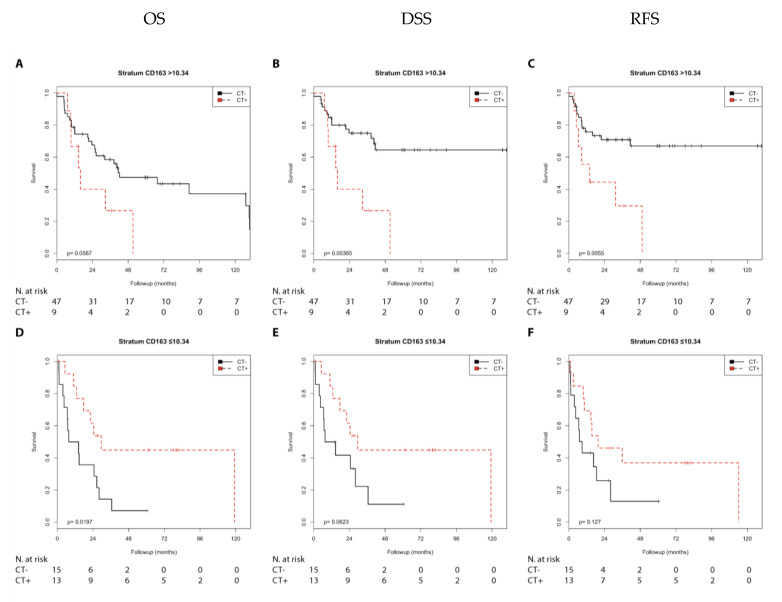
Kaplan–Meier analysis: Association of chemotherapy with prognosis (OS, DSS, and RFS) in the N0/N1 + 2 groups stratified by CD163 expression (>10.34 or ≤10.34). In the N0 group (**A**–**C**) with CD163 (>10.34), chemotherapy was not associated with OS (**A**) but was associated with shorter DSS (**B**; *p* = 0.004) and shorter RFS (**C**; *p* = 0.006). In the N1 + 2 group (**D**–**F**) with CD163 (≤10.34), chemotherapy was associated with longer OS (**D**; *p* = 0.020), but not with DSS (**E**) or RFS (**F**).

In the Kaplan–Meier analysis, chemotherapy was associated with a shorter DSS (*p* = 0.004) and shorter RFS (*p* = 0.006) compared with no chemotherapy and showed a trend with a shorter OS (*p*= 0.057) in the N0 group with high CD163 expression. In contrast, chemotherapy was associated with a longer OS (*p* = 0.020) and showed a trend with a longer DSS (*p* = 0.062) in the N1 + 2 group with low CD163 expression (Supplementary Table S5; Figure 7).

**Figure 8:**
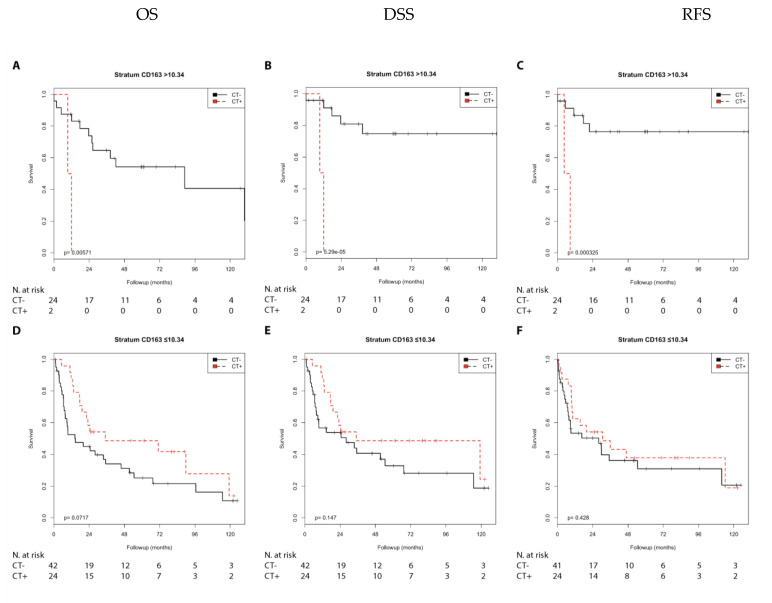
Kaplan–Meier analysis: Association of chemotherapy with prognosis (OS, DSS, and RFS) in the pT2/pT3 + 4 groups stratified by CD163 expression (>10.34 or ≤10.34). In the pT2 group (**A**–**C**) with CD163 (>10.34), chemotherapy was associated with shorter OS (**A**; *p* = 0.006), shorter DSS (**B**; *p* < 0.001), and shorter RFS (**C**; *p* < 0.001). In the pT3 + 4group (**D**–**F**) with CD163 (≤10.34), chemotherapy was not associated with OS (**D**), DSS (**E**), or RFS (**F**).

Chemotherapy was associated with a shorter OS (*p* = 0.006), DSS, and RFS (both *p* < 0.001) compared with no chemotherapy in the pT2 group with high CD163 expression. However, chemotherapy was not associated with prognosis in the pT3 + 4 group with high CD163 expression. In addition, chemotherapy was associated with a trend toward longer OS compared with no chemotherapy (*p* = 0.072) in the pT3 + 4 group with low CD163 expression (Supplementary Table S5; Figure 8).

Next, we performed univariate Cox’s regression analysis. Chemotherapy was associated with a shorter DSS (RR = 3.62; *p* = 0.007), RFS (RR = 3.99; *p* = 0.009), and as a trend with a shorter OS (RR = 2.25; *p* = 0.064) compared with no chemotherapy in the N0 group with high CD163 expression. In contrast, chemotherapy was associated with a longer OS (RR = 0.35; *p* = 0.025) and as a trend with a longer DSS (RR = 0.41; *p* = 0.071) in the N1 + 2 group with low CD163 expression (Table 2).

Chemotherapy was associated with a shorter OS (RR = 8.35; *p* = 0.021), DSS (RR = 27.55; *p* = 0.007), and shorter RFS (RR = 15.35; *p* = 0.007) compared with no chemotherapy in the pT2 group with high CD163 expression. However, chemotherapy was not associated with the prognosis in the pT3 + 4 group with high CD163 expression. In addition, chemotherapy was associated with a trend toward a longer OS (RR = 0.57; *p* = 0.075) compared with no chemotherapy in the pT3 + 4 group with low CD163 expression (Table 3).

In summary, the immune markers further stratified the survival characteristics of patients receiving or not receiving chemotherapy when they were stratified into groups based on the nodal stage and tumor stage.

On the one hand, in the N0 group, patients with high expression of the immune markers CCL2 (RFS), CD68 (DSS and RFS), and CD163 (DSS and RFS) did not benefit from chemotherapy. Likewise, in patients in the pT2 group with high expression of the immune markers CCL2 (OS, DSS, and RFS), CD68 (DSS, and RFS), and CD163 (OS, DSS, and RFS), chemotherapy was not associated with a better prognosis.

On the other hand, in the N1 + 2 group, patients with low expression of the immune markers CCL2 (DSS and RFS), CD68 (OS, DSS, and RFS), and CD163 (OS) saw a significant benefit from receiving chemotherapy. Similarly, patients with pT3 + 4 tumors and a low expression of the immune marker CD68 (OS) had a benefit from receiving chemotherapy, and a trend toward an association with improved OS was seen for patients with a low expression of CD163.

CD68 appeared to be the most applicable immune marker to stratify the outcome of chemotherapy for patients categorized by the nodal stage and tumor stage.

### 2.3. Association of Chemotherapy with Prognosis in Different Nodal Stage and Tumor Stage Groups (Adjusted for CCL2, CD68, and CD163 Expression) in Muscle Invasive Bladder Cancer Patients

To study whether the association of chemotherapy with the prognosis was independent from the immune markers in the different nodal stage and tumor stage patient groups, we performed multivariate Cox’s regression analyses (adjusted for the immune markers CCL2, CD68, and CD163). Chemotherapy treatment was in none of the nodal stage/tumor stage groups (N0/T2; N0/T3 + 4; N1 + 2/T2; and N1 + 2/T3 + T4) an independent marker for prognosis (Table 4). There was only a trend toward significance in the N1 + 2/T3 + 4 group for OS and DSS with chemotherapy (*p* = 0.058 and *p* = 0.067) and in the N0/T3 + 4 for RFS without chemotherapy (*p* = 0.099).

Altogether, the multivariate Cox’s regression analysis suggested that the association between chemotherapy and prognosis depended on the immune cells. Next, examined whether the immune markers in the multivariate Cox’s regression analyses were independently associated with the prognosis in the four nodal stage/tumor stage groups (Table 5). In the N0/T2 group, high CCL2 expression showed a trend associated with a longer DSS (RR = 0.20; *p* = 0.095) and longer RFS (RR = 0.21; *p* = 0.099). In the N0/T3 + 4 group, again, high CCL2 expression was associated only as a trend with longer DSS (RR = 0.41; *p* = 0.054) and longer RFS (RR = 0.43; *p* = 0.051).

In the same group, high CD68 expression was associated with longer RFS (RR = 0.35; *p* = 0.027) and as a trend with longer OS (RR = 0.49; *p* = 0.072) and longer DSS (RR = 0.41; *p* = 0.057), whereas high CD163 expression was associated with shorter OS (RR = 2.44; *p* = 0.016), DSS (RR = 3.02; *p* = 0.008), and RFS (RR = 3.25; *p* = 0.007). In the N1 + 2/T2 group, high CD163 expression was associated as a trend with a shorter RFS (RR = 14.32; *p* = 0.080). In the N1 + 2/T3 + 4 group, high CCL2 expression was associated with a shorter OS (RR = 5.38; *p* = 0.001), DSS (RR = 4.12; *p* = 0.016), and RFS (RR = 4.26; *p* = 0.011), but high expression of CD68 was associated as a trend with a longer OS (RR = 0.43; *p* = 0.088).

Altogether, the prognostic value of CCL2 expression, as reported previously [13], was dependent on the nodal stage, here either as a trend associated with a good prognosis in the two N0 groups or with poor prognosis in the N1 + 2 (T3 + 4) group. CD68 as a pan-marker of macrophages appeared to be associated at least as a trend with better prognosis in the N0 (T2) and an N1 (T3 + 4) group. In contrast to this, CD163 as M2 macrophage marker appeared to be associated significantly with poor prognosis in the N0 (T3 + 4) group and as a trend in the N1 + 2 (T2) group.

## 3. Discussion

Others and we have shown that the tumor immune microenvironment is associated with the prognosis [7,8,9,10,11]. High stromal tumor infiltrating leukocytes indicated an inflamed subtype with an 80% 5-year DSS, and a lack of immune infiltrates identified an uninflamed subtype with a survival rate of less than 25% in muscle invasive BCa [9].

On the other hand, the presence of tumor-associated macrophages is associated with a poor prognosis in different cancers, including BCa [22,23]. How can these seemingly contradictory findings be reconciled?

A major part of the immune microenvironment, apart from T-cells and natural killer cells, is macrophages [9], and we were interested in whether the presence of macrophages could be associated with the prognosis of BCa patients. To detect macrophages, we employed the pan-macrophage markers CD68, CD163 (M2-macrophages), and CCL2, which are known to be expressed by the monocyte-macrophage lineage [24,25]. Since the tumor stage (pT) and lymph node stage (pN) are well accepted as prognostic factors for bladder cancer patients treated with radical cystectomy [4,5,6], we also included these factors in our study.

A major but still open clinical question is whether patients treated with adjuvant chemotherapy always have a better prognosis than those not treated with adjuvant chemotherapy. In other words, is it possible to predict which patients will benefit from chemotherapy in terms of prognosis? First, we considered the clinicopathological factors of the pathological tumor stage (pT2 vs. pT3 + 4) and lymph node stage (N0 vs. N1 + 2) to stratify the patients for prognosis analysis. Patients treated with chemotherapy in the N0 patient group showed a shorter RFS while patients in the N1 + 2 group showed a longer OS than patients not treated with chemotherapy. Accordingly, patients treated with chemotherapy in the pT2 group showed a shorter RFS and shorter DSS than patients not treated with chemotherapy (though it should be noted that this group had a small number of patients); however, there was no difference between patients treated with or without chemotherapy in the pT3 + 4 group.

Next, we were interested in whether similar prognostic patterns could be determined by the presence of macrophages as identified by CD68, CD163, or CCL2 staining.

On the one hand, chemotherapy was not beneficial in patients in the N0 group with high expression of the immune markers CCL2 (RFS), CD68 (DSS and RFS), and CD163 (DSS and RFS) compared to no chemotherapy. Similarly, chemotherapy also had no survival benefit in patients in the pT2 group with high expression of the immune markers CCL2 (OS, DSS, and RFS), CD68 (OS, DSS, and RFS), and CD163 (OS, DSS, and RFS). Again, this group had a small number of patients. A patient selection for chemotherapy at the N1/2 stage disease is expected, although in our study in the pT2 group out of the seven N1/2 stage patients four received chemotherapy and three did not.

On the other hand, chemotherapy was associated with a better prognosis in patients in the N1 + 2 group with low expression of the immune markers CCL2 (DSS and RFS), CD68 (OS, DSS, and RFS), and CD163 (OS) compared with no chemotherapy. Similarly, chemotherapy was associated with a better prognosis in patients in the pT3 + 4 group with low expression of the immune marker CD68 (OS and DSS). Altogether, the pan macrophage marker CD68 showed more frequent associations with prognosis than did the markers CD163 and CCL2.

Considering a higher expression of macrophage markers as a surrogate for a higher presence of macrophages, we suggest that an immune microenvironment rich in macrophages is good for the prognosis. This finding supports our previous result that the presence of tumor-infiltrating immune cells is associated with a superior prognosis [9]. We summarize that patients with a higher expression of macrophage markers treated with chemotherapy showed a poorer prognosis (N0 and pT2 groups) compared with patients with a lower expression of macrophage markers or received no effect from chemotherapy (N1 + 2 and pT3 + 4 groups). However, patients in the N1 + 2 and pT3 + 4 groups with a low expression of macrophage markers showed a better prognosis when treated with chemotherapy compared with those not treated with chemotherapy.

In this way, the characterization of macrophage markers may help to predict how chemotherapy is associated with the prognosis of bladder cancer patients. This could help to spare patients from unnecessary treatments, including the undesirable side effects of chemotherapy, and also prevent missing the opportunity for chemotherapy in patients who are likely to benefit in terms of prognosis. The issue of overtreatment with chemotherapy has been discussed comprehensively in breast cancer [26].

There are molecular markers in addition to the nodal stage status that may allow the stratification of breast cancer patients for a response to chemotherapy and, thus, reduce its application [26]. The 21-gene recurrence score assay can quantify the likelihood of distant recurrence in women with estrogen receptor-positive, lymph node-negative breast cancer treated with adjuvant tamoxifen, and, in addition, this score can predict the magnitude of the chemotherapy benefit [27]. However, such biomarkers are still in their infancy for bladder cancer.

Intriguingly there are several reports in which the expression of different biomarkers was shown to be associated with the response to chemotherapy in bladder cancer patients. An interesting general model for the characterization of predictive factors in MIBC treatment based on key signaling pathways was reported [28]. Next, we discuss single parameters that are predictive for adjuvant chemotherapy in more detail.

The role of MIB-1 (MKI67/Ki67) in predicting the response to adjuvant chemotherapy is controversial. Increased Ki67 staining in lymph node-positive bladder cancer patients treated with adjuvant chemotherapy was associated with decreased disease-specific survival [6]. The authors suggested that increased Ki67 staining could be associated with the development of metastasis after adjuvant chemotherapy, which may reflect a reduced sensitivity to adjuvant chemotherapy in high Ki67-expressing BCa [6]. However, two reports showed that a higher Ki67 labeling index (>8.8% or ≥20%) was associated with a favorable outcome in MIBC patients treated with radiochemotherapy without cystectomy [29,30].

Our hypothesis might be supported by reports concerning the expression of the AT-rich interaction domain-containing protein 1B (ARID1B), a component of the human SWI/SNF chromatin remodeling complex. ARID1B was associated with a poor outcome, and a benefit of adjuvant chemotherapy was observed in patients with low ARID1B expression but not in those with high expression [31]. The reason for this could be that cancer cells lacking ARID1A and ARID1B expression are deficient in DNA repair and potentially vulnerable to DNA damage [31].

In line with this, a clinical benefit from adjuvant chemotherapy was detected in patients with a low expression of multi drug resistance protein 1 (MDR1/synonymous ABCB1), a drug-transport pump protein, and in patients with a low expression of excision repair, complementing defective, in Chinese hamster, (ERCC1), a DNA damage repair protein [32,33]. In the same fashion, forkhead box M1 (FOXM1) is known to regulate the transcription of various DNA repair factors, and its upregulation has been associated with chemotherapy resistance in different cancers, including bladder cancer, which suggests FOXM1 as a promising target to overcome chemotherapy resistance [34,35]. Altogether, defects in DNA repair increased the accessibility of cells/DNA for chemotherapeutics and attenuated the transport of chemotherapeutics out of tumor cells, which can help to predict the benefit of the application of chemotherapy.

In line with these findings, we propose that a depleted immune microenvironment, here represented as a reduction in or loss of macrophages as identified by the markers CD68, CD163, and CCL2, can help to estimate a benefit of chemotherapy in N1 + 2 stage patients. There are, of course, other therapy-relevant immune cells. Wang et al. reported that high infiltration of specific immune cells, i.e., interleukin 17A (IL17A)+ cells comprising CD4 + T helper cells, cytotoxic CD8+ T cells, and γδT cells, can predict the benefit from adjuvant chemotherapy for MIBC patients [12].

However, our study has several limitations. First, this was a retrospective study, and the size of the investigated cohort was limited. Second, we concentrated only on three immune/macrophage markers, which is not fully representative for the complete immune microenvironment. Therefore, prospective large cohort studies are necessary in the future. We think that macrophages play an essential part in the host–tumor interaction and in the therapy response. Tumor-associated macrophages are a plastic and heterogeneous cell population of the tumor microenvironment that can account for up to 50% of some malignancies [36].

About 10 years ago, the group of Weissman showed that bladder tumor-initiating cells, which are prognostically relevant, expressed more CD47 compared with the rest of the tumor. CD47 is a protein that provides an inhibitory signal for macrophage phagocytosis [37]. Recently, others and we showed that immunological signatures on the RNA and/or protein level were prognosis relevant [7,8,9,10,38,39]. However, currently, in clinical practice, only 3–5 markers can be routinely assessed. Therefore, the study and confirmation of single markers is eligible and necessary. Altogether, the role of different immune cells as predictive markers for therapy responsiveness, including chemo-, but also immunotherapy, remains to be studied in more detail.

## 4. Materials and Methods

### 4.1. Patients and Tumor Material

Tissue microarrays (TMAs) with formalin-fixed and paraffin-embedded tumor samples from 168 muscle invasive BCa patients were investigated in this study as previously described (Eckstein et al., 2020). The TMAs were prepared as follows: hematoxylin and eosin stained slides were scanned (Panoramic P250, 3DHistech, Budapest, Hungary) and annotated using a TMA annotation tool (Caseviewer v2). Four cores (diameter 1 mm; two cores from the invasion margin and two cores from the tumor center) were taken utilizing an automated tissue microarray (TMA Grandmaster, 3DHistech) as described previously [40,41].

The tumor histology was reviewed by two uropathologists (AH and ME). An overview of the clinicopathological parameters of the patients included in this study was previously described [13] and is given in Supplementary Tables S1 and S2.

### 4.2. Immunohistochemistry

For the study of CCL2 protein expression, a manual IHC protocol was applied as previously described [13,42]. Briefly, after heat pretreatment at 120 °C for 5 min with tris-ethylenediaminetetraacetic acid (TE)–buffer pH 9 and peroxidase blocking (Dako, Hamburg, Germany), primary antibody against CCL2 (monoclonal mouse IgG1, clone 2D8, Cat.-No. AM06749PU-N, dilution 1:15,000; Acris Antibodies, Herford, Germany) was applied for 30 min. The slides were counterstained for 1 min with hematoxylin (Merck, Darmstadt, Germany). Between all of the steps, the slides were washed with buffer from Dako, and all of the incubation steps were performed at room temperature.

For the study of CD68 and CD163 proteins, staining was performed on a fully automated Ventana Benchmark Ultra autostainer (Ventana, Tucson, AZ, USA) as previously described [13]. Briefly, sections were deparaffinized, and antigens were retrieved by heating the sections in a pH 8.4 tris/borate/ethylenediaminetetraacetic acid (EDTA) solution (Ventana). Endogenous peroxidase was blocked with 1% H_2_O_2_. Visualization of the bound antibody was performed using the ultraVIEW TM DAB system (Ventana). All sections were counterstained with hematoxylin II/Mayer’s hematoxylin (Ventana). IHC staining was performed with antibodies against the following markers: CD68 (mouse monoclonal immunoglobulin G (IgG), clone PG-M1, Thermo Fisher Scientific, dilution 1:60) and CD163 (mouse monoclonal IgG, clone 10D6, Novocastra/Leica, Wetzlar, Germany, dilution 1:500). Immune cells were scored by two pathologists (AH and ME).

Stained specimens were viewed at objective magnifications of ×100 and ×200. Negative control slides without the addition of primary antibody were included for each staining experiment. From each sample, two cores from the center and two cores from the invasive front were analyzed. Afterward, the staining average of both cores was determined as we did not see significant differences between the two locations. The expression of CCL2 was detected in ICs (the average of stained ICs in the invasion front and the tumor center) as the percentage of CCL2-positive ICs out of all ICs. There were no relevant differences in the staining intensities so only the percentage of CCL2-positive ICs was considered.

For the survival analysis, patients were grouped as having ≤6% CCL2 positive ICs vs. >6% CCL2-positive ICs. For the survival analysis, patients were grouped by the median (≤median vs. >median) of the log2-transformed expression for CD68 and CD163. Slides were scanned with a P250 slide scanner (3DHistech, Budapest, Hungary) and analyzed using CaseViewer2.0 (3DHistech).

### 4.3. Immune Cell Quantification via Definiens Developer Software

CD68+ and CD163+ ICs were quantified (counts per mm^2^) and log2-transformed for further analysis using the Definiens Developer Software as described previously [9].

### 4.4. Statistical Analyses

The associations of the expression of CD68, CD163, or CCL2 with the OS, DSS, and RFS were determined through Kaplan–Meier analysis and by univariate and multivariate Cox’s regression analyses.

A *p*-value of less than 0.05 was considered statistically significant. Statistical analyses were performed with the SPSS 21.0 software package (SPSS Inc., Chicago, IL, USA) and with R V3.2.1 (The R foundation for statistical computing, Vienna, Austria).

## 5. Conclusions

The presence of tumor-infiltrating immune cells, here represented as macrophages characterized by the markers CD68, CD163, and CCL2, was associated with a superior prognosis, and chemotherapy may not add an advantage for prognosis. However, a depleted immune microenvironment, here represented as a reduction or loss of macrophages, predicted a benefit of chemotherapy in N1 + 2 stage patients. CD68 appeared to be the most applicable immune marker to stratify chemotherapy outcomes in patients with BCa categorized by nodal stage and tumor stage.

## Figures and Tables

**Table 1:** Univariate Cox’s regression analysis: Association between chemotherapy and prognosis stratified by the nodal stage and/or tumor stage.

	CT+ vs. CT−	Overall Survival (OS)		Disease-Specific Survival (DSS)		Recurrence-Free-Survival (RFS)		
		RR (95%CI)	*p*-Value	RR (95%CI)	*p*-Value	RR (95%CI)	*p*-Value	
Nodal stage								
N0		n.s.	n.s.	1.82 (0.93–3.59)	(0.082)	2.03 (1.05–3.94)	0.035	
N1 + 2		0.52 (0.28–0.99)	0.046	0.55 (0.28–1.11)	(0.097)	n.s.	n.s.	
NX		n.s.	n.s.	n.s.	n.s.	n.s.	n.s.	
Tumor stage								
pT2		n.s.	n.s.	4.10 (1.29–13.04)	0.016	4.61 (1.43–14.84)	0.010	
pT3 + 4		n.s.	n.s.	n.s.	n.s.	n.s.	n.s.	
Combination of nodal and tumor stage								
N0/T2		6.21 (0.72–53.18)	(0.096)	10.28 (1.07–98.98)	0.044	10.47 (1.09–100.70)	0.042	
N0/T3 + 4		n.s.	n.s.	n.s.	n.s.	n.s.	n.s.	
N1 + 2/T2		n.s.	n.s.	n.s.	n.s.	n.s.	n.s.	
N1 + 2/T3 + 4		0.48 (0.24–0.97)	0.042	0.48 (0.22–1.05)	(0.066)	n.s.	n.s.	

**Table 2:** Univariate Cox’s regression analysis: Association between chemotherapy, considering the immune markers CCL2, CD68, and CD163, and prognosis stratified by the nodal stage.

	CT+ vs. CT−	OS		DSS		RFS	
	Parameter	RR (95%CI)	*p*-Value	RR (95%CI)	*p*-Value	RR (95%CI)	*p*-Value
Nodal stage							
N0	CCL2 ≤ 6%	n.s.	n.s.	n.s.	n.s.	n.s.	n.s.
N0	CCL2 > 6%	n.s.	n.s.	n.s.	n.s.	3.33 (0.96–11.51	(0.057)
N0	CD68 ≤ 10.4	n.s.	n.s.	n.s.	n.s.	n.s.	n.s.
N0	CD68 > 10.4	n.s.	n.s.	3.38 (1.22–9.33)	0.019	3.97 (1.48–10.61)	0.006
N0	CD163 ≤ 10.34	n.s.	n.s.	n.s.	n.s.	n.s.	n.s.
N0	CD163 > 10.34	2.25 (0.95–5.32)	(0.064)	3.62 (0.28–9.17)	0.007	3.99 (0.29–8.51)	0.009
N1 + 2	CCL2 ≤ 6%	0.50 (0.22–1.13)	(0.096)	0.39 (0.16–0.95)	0.038	0.41 (0.17–0.96)	0.041
N1 + 2	CCL2 > 6%	n.s.	n.s.	n.s.	n.s.	n.s.	n.s.
N1 + 2	CD68 ≤ 10.4	0.39 (0.16–0.97)	0.042	0.35 (0.13–0.94)	0.036	0.34 (0.13–0.88)	0.026
N1 + 2	CD68 > 10.4	n.s.	n.s.	n.s.	n.s.	n.s.	n.s.
N1 + 2	CD163 ≤ 10.34	0.35 (0.14–0.88)	0.025	0.41 (0.16–1.08)	(0.071)	n.s.	n.s.
N1 + 2	CD163 > 10.34	n.s.	n.s.	n.s.	n.s.	n.s.	n.s.

Abbreviations: 95%CI: 95% confidence interval, RR: relative risk, n.s.: not significant. *p*-values with a trend for significance (*p* > 0.05 and <0.10) are in parentheses.

**Table 3:** Univariate Cox’s regression analysis: Association between chemotherapy, considering the immune markers CCL2, CD68, and CD163, and prognosis stratified by the tumor stage.

	CT+ vs. CT−	OS		DSS		RFS	
	Parameter	RR (95%CI)	*p*-Value	RR (95%CI)	*p*-Value	RR (95%CI)	*p*-Value
Tumor stage							
pT2	CCL2 ≤ 6%	n.s.	n.s.	n.s.	n.s.	n.s.	n.s.
pT2	CCL2 > 6%	11.56 (1.60–83.37)	0.015	24.61 (2.18–277.50)	0.009	25.51 (2.26–288.20)	0.009
pT2	CD68 ≤ 10.4	n.s.	n.s.	n.s.	n.s.	n.s.	n.s.
pT2	CD68 > 10.4	7.39 (0.77–71.13)	(0.083)	21.91 (1.37–351.30)	0.029	22.98 (1.44–367.60)	0.027
pT2	CD163 ≤ 10.34	n.s.	n.s.	n.s.	n.s.	n.s.	n.s.
pT2	CD163 > 10.34	8.35 (1.38–50.52)	0.021	27.55 (2.44–310.40)	0.007	15.35 (2.12–111.20)	0.007
pT3 + 4	CCL2 ≤ 6%	n.s.	n.s.	n.s.	n.s.	n.s.	n.s.
pT3 + 4	CCL2 > 6%	n.s.	n.s.	n.s.	n.s.	2.52 (1.08–5.84)	0.032
pT3 + 4	CD68 ≤ 10.4	0.51 (0.26–0.99)	0.049	0.48 (0.23–1.0)	(0.053)	0.54 (0.27–1.10)	(0.091)
pT3 + 4	CD68 > 10.4	n.s.	n.s.	n.s.	n.s.	2.14 (1.07–4.24)	0.031
pT3 + 4	CD163 ≤ 10.34	0.57 (0.31–1.06)	(0.075)	n.s.	n.s.	n.s.	n.s.
pT3 + 4	CD163 > 10.34	n.s.	n.s.	n.s.	n.s.	n.s.	n.s.

Abbreviations: 95%CI: 95% confidence interval, RR: relative risk, n.s.: not significant. *p*-values with a trend for significance (*p* > 0.05 and <0.10) are in parentheses.

**Table 4:** Multivariate Cox’s regression analysis: Association between chemotherapy and prognosis stratified by nodal stage and tumor stage (adjusted for the immune markers CCL2, CD68, and CD163).

	CT+ vs. CT−		OS		DSS		RFS	
		*N*	RR (95%CI)	*p*-Value	RR (95%CI)	*p*-Value	RR (95%CI)	*p*-Value
N0/T2		35	4.46 (0.45–41.57)	(0.200)	5.24 (0.48–57.48)	(0.175)	5.09 (0.19–55.48)	(0.182)
N0/T3 + 4		69	1.24 (0.64–2.43)	(0.523)	1.60 (0.75–3.42)	(0.226)	1.88 (0.89–3.99)	(0.099) *
N1 + 2/T2		7	0.71 (0.04–11.79)	(0.809)	0.71 (0.04–11.79)	(0.809)	3.21 (0.15–70.80)	(0.459)
N1 + 2/T3 + 4		42	0.48 (0.23–1.03)	(0.058) *	0.47 (0.21–1.06)	(0.067) *	0.65 (0.29–1.44)	(0.286)

Abbreviations: 95%CI: 95% confidence interval, RR: relative risk. All *p*-values were not significant but when they showed a trend towards significance, they are marked by *.

**Table 5:** Multivariate Cox’s regression analysis: Association between immunological markers and prognosis stratified by nodal stage and tumor stage (adjusted for chemotherapy and the immune markers CCL2, CD68, and CD163).

	Immunological Marker		OS		DSS		RFS	
		*N*	RR (95%CI)	*p*-Value	RR (95%CI)	*p*-Value	RR (95%CI)	*p*-Value
N0/T2	CCL2 > 6% vs. ≤6%	35	n.s.	n.s.	0.20 (0.75–31.76)	(0.095)	0.21 (0.74–31.18)	(0.099)
N0/T3 + 4	CCL2 > 6% vs. ≤6%	69	n.s.	n.s.	0.41 (0.17–1.02)	(0.054)	0.43 (0.18–1.00)	(0.051)
N0/T3 + 4	CD68 > 10.4 vs. ≤10.4	69	0.49 (0.22–1.07)	(0.072)	0.41 (0.17–1.03)	(0.057)	0.35 (0.14–0.88)	0.027
N0/T3 + 4	CD163 > 10.34 vs. ≤10.34	69	2.44 (1.18–5.02)	0.016	3.02 (1.33–6.86)	0.008	3.25 (1.37–7.69)	0.007
N1 + 2/T2	CD163 > 10.34 vs. ≤10.34	7	n.s.	n.s.	n.s.	n.s.	14.3 (0.73 –282.72)	(0.080)
N1 + 2/T3 + 4	CCL2 > 6% vs. ≤6%	42	5.38 (1.92–15.03)	0.001	4.12 (1.31–12.98)	0.016	4.26 (1.40–12.93)	0.011
N1 + 2/T3 + 4	CD68 > 10.4 vs. ≤10.4	42	0.43 (0.17–1.13)	(0.088)	n.s.	n.s.	n.s.	n.s.

Abbreviations: 95%CI: 95% confidence interval, RR: relative risk. *p*-values were either significant or when they showed a trend towards significance they are in parentheses.

## Data Availability

Data is contained within this article and the supplementary materials.

## Supplementary Materials

The following are available online at https://www.mdpi.com/2073-4409/10/1/159/s1, Figure S1: Immunohistochemical staining for CD68 and CD163; Table S1: Clinicopathological data and survival parameters of the BCa patients; Table S2A: Overview for the pT/pN stages and chemotherapy of the BCa patients; Table S2B. Correlation of the pT/pN stages and chemotherapy of the BCa patients; Table S2C. Correlation of the pT/pN stages and immune markers of the BCa patients; Table S3: Kaplan–Meier analysis: The association between chemotherapy and prognosis, stratified by nodal stage or tumor stage; Table S4: Kaplan–Meier analysis: The association between chemotherapy and prognosis, stratified by nodal stage and tumor stage; Table S5: Kaplan–Meier analysis: The association between chemotherapy, considering the immune markers CCL2, CD68, and CD163, and prognosis stratified by the nodal stage or tumor stage.
