# Supplementary material for: Immune Cell-Associated Protein Expression Helps to Predict Survival in Muscle-Invasive Urothelial Bladder Cancer Patients after Radical Cystectomy and Optional Adjuvant Chemotherapy
Source: Cells. 2021 Jan 15;10(1):159. doi: 10.3390/cells10010159 (PMC7829767; doi:10.3390/cells10010159)
Supplement: Supplementary file 1 [file cells-10-00159-s001.pdf]

Article

Immune Cell-Associated Protein Expression Helps to Predict Survival in Muscle-Invasive Urothelial Bladder Cancer Patients after Radical Cystectomy and Optional Adjuvant Chemotherapy

Helge Taubert [1,*,†,‡], Markus Eckstein [2,†,‡], Elena Epple [1,2], Rudolf Jung [2], Katrin Weigelt [1], Verena Lieb [1], Danijel Sikic [1,‡], Robert Stöhr [2,‡], Carol Geppert [2], Veronika Weyerer [2], Simone Bertz [2], Astrid Kehlen [3], Arndt Hartmann [2,‡], Bernd Wullich [1,‡] and Sven Wach [1,‡]

[1] Department of Urology and Pediatric Urology, University Hospital Erlangen, FAU Erlangen-Nürnberg, 91054 Erlangen, Germany; elena.epple@fau.de (E.E.); Katrin.Weigelt@uk-erlangen.de (K.W.); Verena.Lieb@uk-erlangen.de (V.L.); danijel.sikic@uk-erlangen.de (D.S.); Bernd.Wullich@uk-erlangen.de (B.W.); sven.wach@uk-erlangen.de (S.W.)

[2] Institute of Pathology, University Hospital Erlangen, FAU Erlangen-Nürnberg, 91054 Erlangen, Germany; markus.eckstein@uk-erlangen.de (M.E.); Rudolf.Jung@uk-erlangen.de (R.J.); robert.stoehr@uk-erlangen.de (R.S.); carol.geppert@uk-erlangen.de (C.G.); veronika.weyerer@uk-erlangen.de (V.W.); simone.bertz@uk-erlangen.de (S.B.); Arndt.Hartmann@uk-erlangen.de (A.H.)

[3] Institute of Medical Microbiology, Medical School, Martin-Luther-University Halle-Wittenberg, 06112 Halle (Saale), Germany; astrid.kehlen@uk-halle.de

* Correspondence: helge.taubert@uk-erlangen.de; Tel.: +49-9131-8542658; Fax: +49-9131-8523374

† Authors contributed equally.

‡ Authors belong to Bridge Consortium, Germany.

Table S1. Clinicopathological data and survival parameters of the BCa patients.

Clinicopathological and Survival Parameters	Patients (Percentage)
Total	168
**Morphology**	
NOS	91 (54.1)
Squamous	37 (22.0)
Sarcomatoid	9 (5.4)
MPUC	9 (5.4)
PUC	6 (3.6)
Pure neuroendocrine	4 (2.4)
Other rare subtypes	12 (7.1)
**Gender**	
female	42 (25)
male	126 (75)
**Age (years)**	
range	41.0–90.0
mean	69.7
median	71.0
**Tumor Stage**	
pT2	44 (26.2)
pT3	85 (50.6)
pT4	39 (23.2)
**Tumor Grade 1973**	
G2	6 (3.6)
G3	162 (96.4)
**Tumor Grade 2004**	

high grade	168
**Nodal Stage**	
pN0	104 (61.9)
pN1 + 2	49 (29.2)
pNX	15 (8.9)
**Adjuvant Chemotherapy (Ct)**	
yes	46 (27.4)
no	122 (72.6)
**Survival/observation Time (months)**	
range	0-135.7
mean	34.5
median	23.9
**Overall Survival (OS)**	
alive	46 (27.4)
dead	122 (72.6)
**Disease-Specific Survival (DSS)**	
alive	78 (46.4)
dead	90 (53.6)
**Relapse-Free Survival Time (months)**	
range	0–135.7
mean	31.0
median	16.1
**Relapse-Free Survival (RFS)**	
without relapse	92 (54.8)
with relapse	76 (45.2)
**Stromal TILs**	
0	11 (6.6)
1–9%	58 (34.5)
10–50%	84 (50.0)
51–80%	13 (7.7)
>80%	2 (1.2)
**Molecular Subtypes**	
Basal	79 (47.0)
DN	4 (2.4)
Luminal	69 (41.1)
Luminal EMT-p53-like	16 (9.5)

Table S2A. Overview for pT/pN stages and Chemotherapy of the BCa patients

		**Chemotherapy**	
**Parameter**	**N**	**yes**	**no**
**pT**	168	46	122
pT2	44	5	39
pT3	85	26	59
pT4	39	15	24
**pN**	168	46	122
pN0	104	21	83
pN1/2	49	21	28
pNX	15	4	11

Table S2B. Correlation of pT/pN stages and chemotherapy of the BCa patients

			pT	pN	Adj. Chemo
Spearman-Rho	pT	correl. coeff.	1	0.261	0.228
		Sig. (2-sided)		**0.001**	**0.003**
	pN	correl. coeff.		1	0.150
		Sig. (2-seitig)			(0.052)

Abbreviations: correl. coeff.: correlation coefficient; Adj. Chemo: Adjuvant chemotherapy; Significant p-values are in bold face. P-value with a trend of significance is in parenthesis.

Table S2C. Correlation of pT/pN stages and immune markers of the BCa patients

Spearman Rho		pT	pN	CCL2	CD68	CD163
pT	correl. coeff.	1	0.261	0.151	–0.015	–0.033
			0.001	0.054	0.851	0.674
pN	correl. coeff.		1	0.097<br>0.217	–0.130<br>0.092	–0.104<br>0.182
CCL2	correl. coeff.			1	–0.130	–0.159*
					0.097	0.042
CD68	correl. coeff.				1	0.468<br><0.001

Abbreviations: correl. coeff.: correlation coefficient; Adj. Chemo: Adjuvant chemotherapy

Significant *p*-values are in bold face. P-value with a trend of significance is in parenthesis.

Table S3. Kaplan-Meier analysis: Association between chemotherapy and prognosis stratified by nodal stage or tumor stage

	CT + vs. CT–	OS	DSS	RFS	Remarks	
		p-value	*p*-value	*p*-value	CT favor.	CT not favor.
**Nodal stage**						
N0		n.s.	(0.078)	0.032		RFS
N1+2		0.042	(0.091)	n.s.	OS	
NX		n.s.	n.s.	n.s.		
**Tumor stage**						
pT2		n.s.	0.010	0.005		DSS, RFS
pT3+4		n.s.	n.s.	n.s.		

Abbreviations: favor.: favorable; not favor.: not favorable; *p*-values with a trend for significance (p>0.05<0.10) are in brackets

Table S4. Kaplan-Meier analysis: Association between chemotherapy and prognosis stratified by nodal stage and tumor stage

	CT+vs.CT-	OS	DSS	RFS	Remarks	
		p-value	*p*-value	*p*-value	CT favor.	CT not favor.
**N0/T2**		(0.056)	0.012	0.011		DSS, RFS
**N0/T3+4**		n.s.	0.036	n.s.		
**N1+2/T2**		n.s.	n.s.	n.s.		
**N1+2/T3+4**		0.036	(0.059)	n.s.	OS	

Abbreviations: favor.: favorable; not favor.: not favorable; *p*-values with a trend for significance ($p > 0.05 < 0.10$) are in brackets

Table S5. Kaplan-Meier analysis: Association between chemotherapy considering the immune markers CCL2, CD68 and CD163 and prognosis stratified by nodal stage or tumor stage

	CT+ vs. CT−	OS	DSS	RFS	Remarks	
	Parameter	*p*-value	*p*-value	*p*-value	CT favor.	CT not favor.
**Nodal stage**						
N0	CCL2≤6%	n.s.	n.s.	n.s.		
N0	CCL2>6%	n.s.	n.s.	0.044		RFS
N0	CD68≤10.4	n.s.	n.s.	n.s.		
N0	CD68>10.4	n.s.	0.012	0.003		DSS, RFS
N0	CD163≤10.34	n.s.	n.s.	n.s.		
N0	CD163>10.34	(0.057)	0.004	0.006		DSS, RFS
N1+2	CCL2≤6%	(0.089)	0.031	0.035	DSS, RFS	
N1+2	CCL2>6%	n.s.	n.s.	n.s.		
N1+2	CD68≤10.4	0.035	0.028	0.020	OS, DSS, RFS	
N1+2	CD68>10.4	n.s.	n.s.	n.s.		
N1+2	CD163≤10.34	0.020	(0.062)	n.s.	OS	
N1+2	CD163>10.34	n.s.	n.s.	n.s.		
**Tumor stage**	Parameter					
pT2	CCL2≤6%	n.s.	n.s.	n.s.		
pT2	CCL2>6%	0.002	<0.001	<0.001		OS,DSS,RFS
pT2	CD68≤10.4	n.s.	n.s.	n.s.		
pT2	CD68>10.4	0.042	0.002	0.001		OS, DSS, RFS
pT2	CD163≤10.34	n.s.	n.s.	n.s.		
pT2	CD163>10.34	0.006	<0.001	<0.001		OS, DSS, RFS
pT3+4	CCL2≤6%	n.s.	n.s.	n.s.		
pT3+4	CCL2>6%	n.s.	n.s.	0.026		RFS
pT3+4	CD68≤10.4	0.044	0.048	(0.086)	OS, DSS	
pT3+4	CD68>10.4	n.s.	n.s.	0.027		RFS

pT3+4	CD163≤10.34	(0.072)	n.s.	n.s.
pT3+4	CD163>10.34	n.s.	n.s.	n.s.

Abbreviations: favor.: favorable; not favor.: not favorable

Suppl. **Figure S1.** Immunohistochemical staining for CD68 and CD163

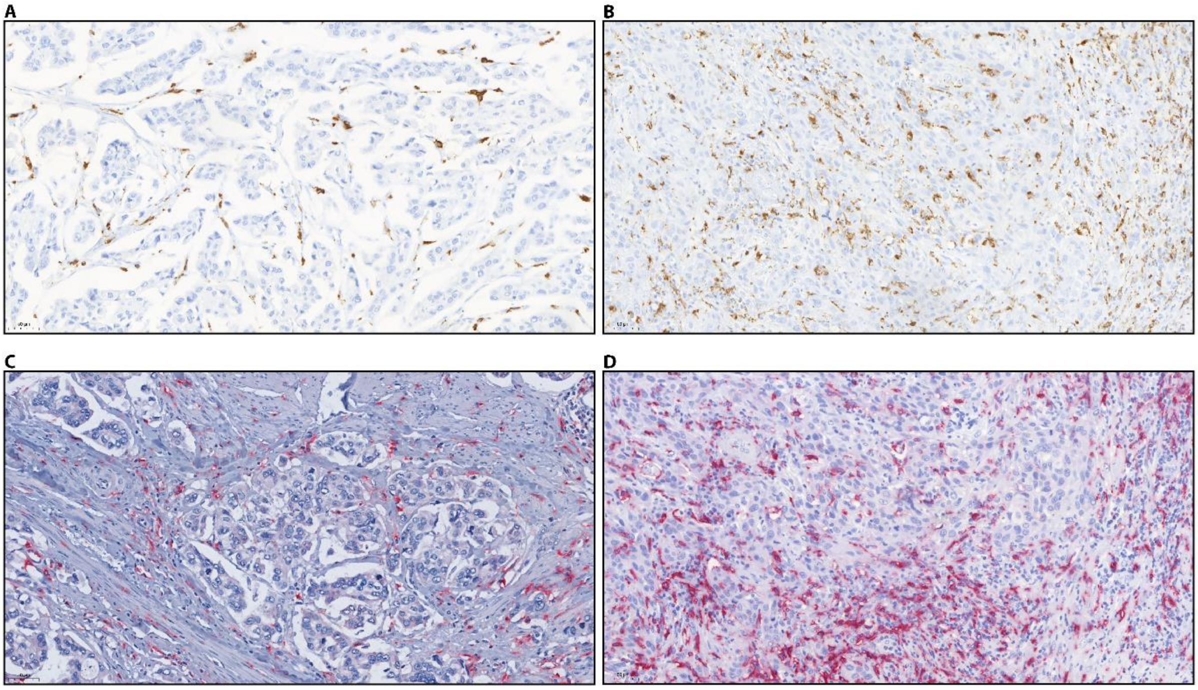

Immunohistochemical staining for CD68 and CD163 in immune cells (macrophages).

Upper row: CD68 staining, (A) low CD68 staining (≤10.4); (B) high CD68 staining (>10.4)

Lower row: CD163 staining, (C) low CD163 staining (≤10.34); (D) high CD163 staining (>10.34). All photos are at 20× magnification.
